# Experimental and computational snapshots of C-C bond formation in a C-nucleoside synthase

**DOI:** 10.1098/rsob.220287

**Published:** 2023-01-11

**Authors:** Wenbo Li, Georgina C. Girt, Ashish Radadiya, James J. P. Stewart, Nigel G. J. Richards, James H. Naismith

**Affiliations:** ^1^ Structural Biology, The Rosalind Franklin Institute, Didcot OX11 0QS, UK; ^2^ Division of Structural Biology, Nuffield Department of Medicine, Roosevelt Drive, Oxford OX3 7BN, UK; ^3^ School of Chemistry, Cardiff University, Park Place, Cardiff CF10 3AT, UK; ^4^ Stewart Computational Chemistry, Colorado Springs, CO 80921, USA; ^5^ Foundation for Applied Molecular Evolution, Alachua, FL 32615, USA

**Keywords:** C-nucleoside, X-ray crystallography, formycin, biosynthesis

## Abstract

The biosynthetic enzyme, ForT, catalyses the formation of a C-C bond between 4-amino-1*H*-pyrazoledicarboxylic acid and MgPRPP to produce a C-nucleoside precursor of formycin A. The transformation catalysed by ForT is of chemical interest because it is one of only a few examples in which C-C bond formation takes place via an electrophilic substitution of a small, aromatic heterocycle. In addition, ForT is capable of discriminating between the aminopyrazoledicarboxylic acid and an analogue in which the amine is replaced by a hydroxyl group; a remarkable feat given the steric and electronic similarities of the two molecules. Here we report biophysical measurements, structural biology and quantum chemical calculations that provide a detailed molecular picture of ForT-catalysed C-C bond formation and the conformational changes that are coupled to catalysis. Our findings set the scene for employing engineered ForT variants in the biocatalytic production of novel, anti-viral C-nucleoside and C-nucleotide analogues.

## 1. Introduction

Driven, in part, by the success of remdesivir in the treatment of viral infections, such as SARS-CoV-2 and Ebola, [1,2] and the development of elegant pro-drug strategies for increasing the bioavailability of functionalized nucleotides, [3,4] there is renewed interest in C-nucleoside derivatives as anti-viral and anti-cancer drugs [5–8]. C-nucleosides and C-nucleotides are, of course, resistant to inactivation by hydrolysis of the sugar base linkage, in contrast with the more common C-N nucleotides [9]. Chemical synthesis of C-nucleosides uses two broad approaches: forming the heterocyclic nucleobase from an acyclic substituent attached to the sugar moiety or coupling a preformed heterocycle to the carbohydrate [10–15]. Both of these strategies often require the use of protecting groups, thereby increasing synthetic complexity. Biocatalysis offers an attractive alternative if we can understand the pathways that give rise to naturally occurring C-nucleosides and C-nucleotides [16–18].

Insights into the biosynthetic origins of bioactive C-nucleosides have resulted from the identification of the gene clusters encoding a number of these secondary metabolites [19–28]. In broad terms, the cellular pathways to these natural products use three distinct chemical strategies to make the key C-C bond (electronic supplementary material, figure S1). In the showdomycin and minimycin pathways, C-glycosidic bond formation is catalysed by the C-glycosynthases SdmA and MinB [24,28,29], respectively, which are both homologous to the glycosidase YeiN [30]. As a result, it is likely that the C-C bond is formed by nucleophilic attack of the double bond in the small molecule substrate on a covalent intermediate that is formed by reaction of the ring-opened sugar with a protein lysine [31]. In the biosynthesis of malayamycin A and pseudouridimycin, a pseudouridine synthase catalyses cleavage of the C-N bond between uridine and ribose-5-phosphate. Re-attachment of the nucleobase to the ribose via a C-C bond then follows rotation of the base within the active site [26,27]. In the third strategy, formycin A and pyrazofurin A arise from direct coupling of a small heteroaromatic dicarboxylic acid to 5′-phosphoribose-1′-pyrophosphate (PRPP) with concomitant decarboxylation [22,30].

**Figure 1.**
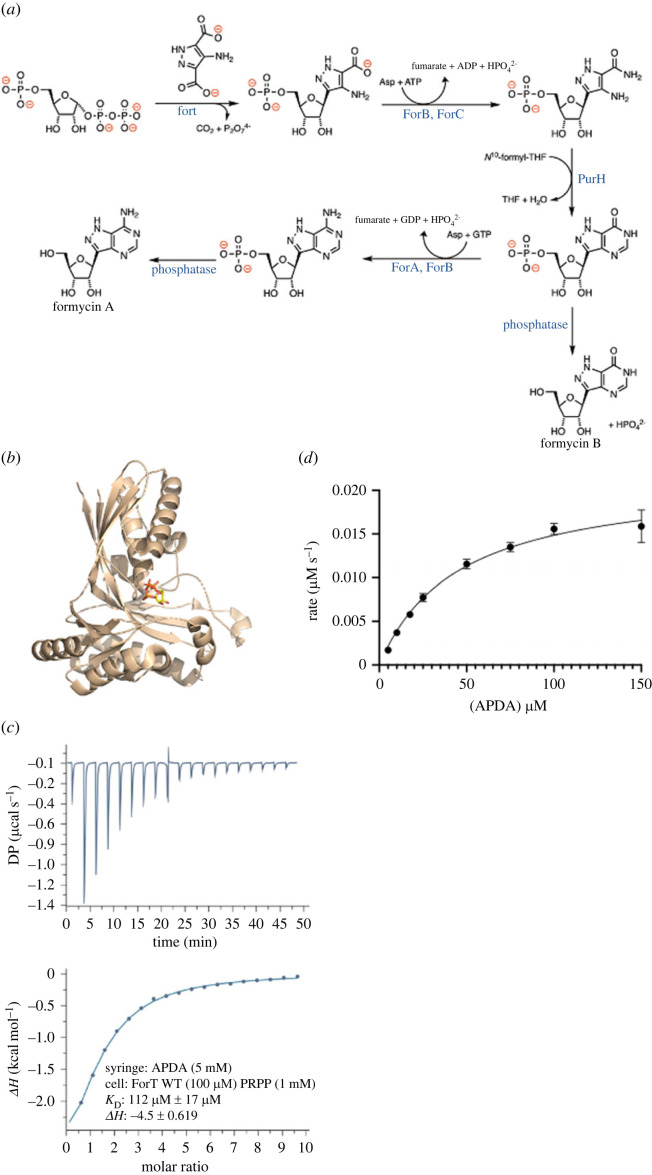
(*a*) The later stages of formycin biosynthesis showing the role of ForT in forming the C-C bond in the C-nucleoside antibiotic. (*b*) The previously reported PRPP ForT structure (RCSB 6YQQ) [32]. The protein is shown in cartoon form and PRPP shown as sticks, with carbon atoms yellow, oxygen red and phosphorus orange. (*c*) ITC (raw and fitted data) shows that ForT binds APDA only in the presence of PRPP, Mg^2+^ is not present. (*d*) Product (CAPR) formation by ForT shows conventional Michaelis–Menten kinetics when APDA is varied while PRPP is held constant at 500 µM.

In the specific case of formycin, the enzyme ForT catalyses C-C bond formation between 4-amino-1*H*-pyrazole-3,5-dicarboxylate (APDA) and PRPP, in a reaction that is driven by the elimination of CO_2_ and inorganic pyrophosphate (PPi) (figure 1*a*) [22,30]. Feeding studies suggest that glutamic acid and lysine are precursors of APDA [33,34], although the molecular transformations in the cell that yield this highly functionalized pyrazole intermediate from hydrzinoglutaric acid [35] are, as yet, unknown. In the final steps of the biosynthetic pathway, the C-riboside product of APDA and PRPP is elaborated by four enzymes to give the pyrazolopyrimidine ring system of formycin A (figure 1*a*) [36].

ForT catalyses C-C bond formation in formycin A biosynthesis (figure 1*a*), and we have previously determined its structure complexed to PRPP (figure 1*b*) [32]. This structure establishes that ForT is a member of the GHMP kinase superfamily [37]. Based on the location of the PRPP binding site, we mutated a number of residues that could plausibly be involved in catalysis and/or substrate binding (table 1). We now report biophysical measurements on these ForT variants, structural studies and semi-empirical (PM7) quantum chemical (QC) calculations that provide a detailed molecular picture of ForT-catalysed C-C bond formation, including the conformational changes coupled to catalysis. Our findings not only confirm previous mechanistic proposals [30,32], but also set the scene for future efforts to employ ForT in the biocatalytic production of novel, anti-viral C-nucleoside and C-nucleotide analogues.

**Table 1.** ITC measurements of binding affinity, in the absence of Mg^2+^, for PRPP by free ForT and APDA to the ForT/PRPP binary complex, and steady-state kinetic parameters (determined by the LC-MS assay) for WT ForT and site-specific ForT variants. Data for the ITC measurements (figure 1*c;* electronic supplementary material, figure S6) and the kinetic assays are shown elsewhere (figure 1*d;* electronic supplementary material, figure S7).

enzyme	PRPP *K*_D_ (μM)	APDA *K*_D_ (μM)	APDA App. *K*_M_ (μM)	*k*_cat_ (s^−^1)
WT ForT	0.93 ± 0.04	110 ± 17	45 ± 10	0.54 ± 0.05
S21A	0.35 ± 0.02	110 ± 40^a^	4300 ± 540	0.061 ± 0.005
L24A	1.5 ± 0.1	>1 mM^a^	1300 ± 180	0.130 ± 0.008
T138A	6.7 ± 0.5	>1 mM^a^	220 ± 21	0.20 ± 0.006
T138V	0.7 ± 0.4	150 ± 82	69 ± 9	0.013 ± 0.007
S174A	6.0 ± 0.3	>1 mM^a^	210 ± 54	0.10 ± 0.008
E211A	0.95 ± 0.02	180 ± 17	inactive^c^	inactive^c^
K263A	20 ± 8	>1 mM^b^	3200 ± 1700	0.21 ± 0.05

^a^Weak binding is observed, and affinity can only be estimated.

^b^No binding under the conditions of the ITC assay.

^c^No detectable product formation.

## 2. Results

### 2.1. Biophysical measurements

Our prior work confirmed the interactions between various active site residues and PRPP. We therefore extended these studies to obtain the binding affinity of APDA to the ForT/PRPP binary complex at room temperature using isothermal calorimetry (ITC) measurements. *K*_D_ values of 0.9 µM and 180 µM were determined for PRPP binding to WT ForT and for APDA binding to the WT ForT/PRPP complex, respectively (figure 1*c* and table 1). All of these ITC studies were, however, performed in the absence of Mg^2+^, conditions where the enzyme is inactive on the timescale of the measurements. The omission of the catalytically important metal ion thereby avoids any heat generated by catalytic turnover ‘contaminating’ heat released by ligand binding. As a result, these ITC studies report only on the affinity of PRPP and APDA for the free WT enzyme and its PRPP binary complex, respectively.

Typical Michaelis–Menten kinetics were observed for freshly purified WT ForT in an LC-MS assay (electronic supplementary material, figure S2) when APDA was varied in the presence of saturating levels of MgPRPP at room temperature (figure 1*d*). Curve-fitting gave an apparent K_M_ for APDA of 45 µM and a turnover number (*k*_cat_) of 0.54 s^−^^1^. The recombinant WT enzyme can catalyse C-C bond formation in the presence of either Mg^2+^ or Mn^2+^ but not Ca^2+^ (electronic supplementary material, figure S3). Little change in activity (within error) is seen over a pH range of 6–8 (electronic supplementary material, figure S4), although the rate of the ForT-catalysed reaction can be increased by using NaH_2_PO_4_/Na_2_HPO_4_ rather than HEPES as the assay buffer (electronic supplementary material, figure S5). In our hands, however, WT ForT loses activity when stored at 4°C in 10 mM HEPES (pH 7.4) containing 150 mM NaCl and 1 mM TCEP. Several of the ForT variants that we have expressed are also prone to precipitation.

Similar biophysical and kinetic studies were performed for a series of ForT variants to explore the effects of replacing conserved, active site residues on ligand binding and catalytic properties (electronic supplementary material, figures S6 and S7). All of the ForT variants exhibited reduced activity but the only one for which we confidently measured no activity was E211A; ITC measurements show that this amino acid substitution does not alter the binding of both substrates (table 1). Of the remaining ForT variants, a 10-fold reduction in *k*_cat_ results from replacing Thr138 by valine (T138V). Perhaps surprisingly, *k*_cat_ is reduced only fourfold in the T138A ForT variant. Both of these substitutions, however, have little impact on the apparent *K*_M_ of APDA (table 1). By contrast, the L24A variant exhibits only a small reduction in *k*_cat_ but a 20-fold reduction in affinity for APDA on the basis of both the *K*_M_ and *K*_D_ values. The T138S and Q266A ForT variants are unstable, preventing any useful binding measurements or kinetic characterization.

### 2.2. Structural biology

**Figure 2.**
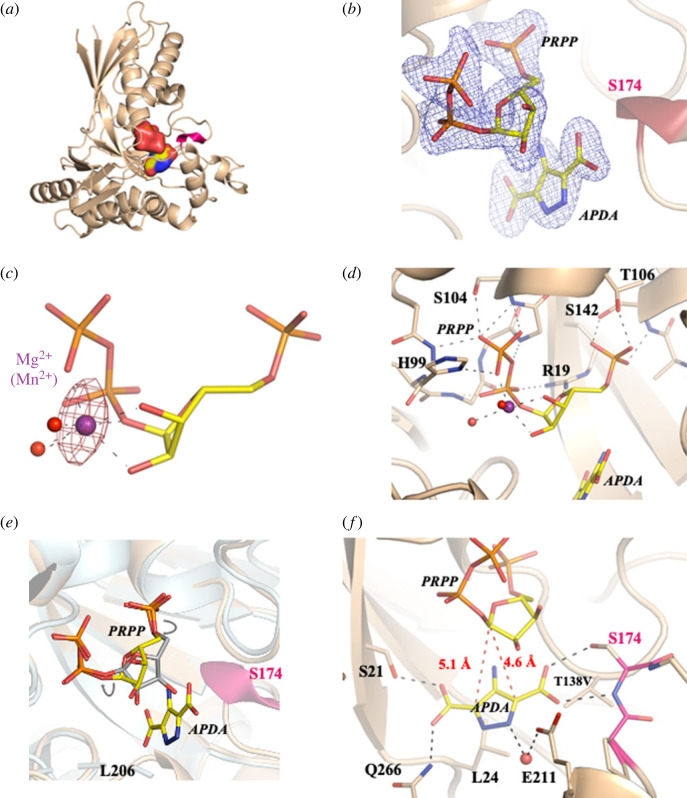
(*a*) The ternary complex structure of the T138V variant is shown in cartoon form (coloured wheat) and the substrates shown as molecular surface. The loop at Ser174 which is ordered in this structure is coloured magenta. The substrates are shown as molecular surfaces, with PRPP sitting above APDA. Carbon atoms are coloured yellow, oxygen atoms coloured red, phosphorous atoms orange and nitrogen atoms blue. (*b*) Fo-Fc omit electron density map (blue mesh) contoured at 3*σ* unambiguously locates PRPP and APDA (shown as stick coloured as in figure 2*a*) in the T138V protein structure. (*c*) The anomalous Fourier map contoured at 3*σ* (red mesh) collected at 9000 eV on Mn^2+^-soaked crystals. The map confirms the location of Mg^2+^ (purple sphere) and its coordination (shown as grey dashed lines) to PRPP (coloured as in figure 2*a*) and two water molecules (red spheres). (*d*) PRPP is anchored to the protein by the interactions of the phosphate groups. Hydrogen bonds are drawn as black dashed lines. Protein carbon atoms are coloured wheat, other atoms are coloured as in figure 2*a*. (*e*) Superposition of the ternary complex and the WT ForT/PRPP binary complex (RCSB 6YQQ) [32] (pale blue cartoon) with carbon atoms of PRPP coloured in grey and other atoms as figure 2*a*. The ribose ring has rotated (denoted by grey arrows) around the two phosphate tethers which are identically positioned in both complexes. The loops at Ser174 and Leu206 are disordered in the binary complex but become ordered by APDA binding. (*f*) One carboxylate group of APDA interacts with Ser21 and Gln266, while the other carboxylate binds to Ser174 thereby ordering the loop. The two possible carbon atoms of APDA which could form the C-C bond are highlighted by a red dashed line to the C1′ of ribose of PRPP.

In carrying out our preliminary analysis of functionally important active site residues [32], we discovered that the T138V variant expressed, purified and crystallized more readily than the WT enzyme. We therefore sought to exploit this variant in structural studies aimed at delineating how both PRPP and APDA were accommodated within the active site. The structure of the T138V/APDA/MgPRPP ternary complex (figure 2*a*) was determined to 1.6 Å in the space group C222_1_ by molecular replacement, using WT ForT (PDB 6YQQ) without PRPP as the search model (table 2) [32]. Bound PRPP and APDA were easily placed in clear difference electron density in the active site (figure 2*b*). Locating the Mg^2+^ ion bound to PRPP, however, proved difficult due to the complex appearance of the electron density. We therefore collected data at the anomalous edge of manganese on crystals of the ternary complex formed when Mn^2+^ rather than Mg^2+^ was present in the crystallization solution. In the resulting anomalous difference Fourier map, the highest peak appeared where we had tentatively positioned Mg^2+^ in our original model (figure 2*c*). In our structure, the Mg^2+^ ion is coordinated in a distorted octahedron to both hydroxyl groups of the ribose, two oxygens in the pyrophosphate moiety and two water molecules (O-Mg^2+^ distances of 2.0–2.2 Å). This coordination in the ternary complex resembles that first seen in glutamine phosphoribosylpyrophosphate amidotransferase, which catalyses formation of 5-phosphoribosyl-1-amine from ammonia and PRPP [38]. A similar arrangement of ligands is also observed for one of the Mg^2+^ ions in the very high-resolution structure of hypoxanthine-guanine phosphoribosyltransferase (HGPRT) [39], the enzyme that catalyses C-N bond formation between PRPP and inosine or guanosine (electronic supplementary material, figure S8). The distorted octahedral arrangement around the Mg^2+^ ion is thought to activate PRPP for catalysis. There is a second Mg^2+^ in HGRPT that coordinates the pyrophosphate group [39], but this is absent in our structure, the space being occupied by the backbone of Gly101 (figure 2*d*). In addition, the alignment of amide dipoles in the backbone about Gly101 allows the negatively charged phosphate group to form favourable charge-dipole interactions with the enzyme.

**Table 2.** Data collection and refinement statistics.

	L24A/CAPR/PPi	T138V/APDA/MgPRPP	T138V/APDA/MnPRPP
beamline	I03 (DLS)	I03 (DLS)	I03 (DLS)
wavelength (Å)	1.3776	0.9762	1.3776
space group	P4_1_2_1_2	C222_1_	C222_1_
a, b, c (Å)	78.9, 78.9, 111.0	66.3, 139.0, 109.6	66.9, 138.7, 109.9
*α*, *β*, *γ* (°)	90, 90, 90	90, 90, 90	90, 90, 90
resolution range (Å)	64.3–1.9 (1.94–1.90)	60–1.60 (1.63–1.60)	53–1.57 (1.60–1.57)
*R*_meas_	0.106 (2.6)	0.065 (3.7)	0.119 (2.5)
I/*σ*(I)	16 (0.6)	15 (0.7)	11 (0.4)
completeness (%)	100 (100)	100(100)	97 (77)
cc1/2	1 (0.9)	1 (0.8)	1 (0.4)
multiplicity	23 (25)	13 (13)	11(5)
no. of reflections	28,350 (1,798)	63,680 (4,624)	756,217 (26,285)
*R*_work_	0.195	0.187	
*R*_free_	0.232	0.213	
number of atoms			
total	2674	2906	
protein	2480	2551	
ligands/ions	23	34	
waters	119	309	
buffer	52	12	
B-factor (Å2)			
protein	27	32	
ligands/ions	54	25	
waters	51	40	
buffer	62	49	
bond lengths (Å)	0.007	0.008	
bond angles (°)	1.44	1.43	
Ramachandran favoured/outlier (%)	97/1	98/1	
molprobity centile	99	98	

The overall protein structure in this ternary complex is little changed from that observed for the enzyme in the WT ForT/PRPP binary complex [32], with an RMSD of only 0.6 Å over 320 overlapping C*_α_* atoms. The interactions of PRPP with the T138V variant, which are dominated by the 5′-phosphate and the pyrophosphate, are essentially unchanged from those in the WT ForT/PRPP binary complex, and the phosphate moieties are found in almost identical positions in the two structures (figure 2*e*). The bound APDA, however, partly occupies space in which ribose was located in the WT ForT/PRPP binary complex [32]. As a result, the PRPP ribose ring in the ternary complex adopts a C2′endo conformer and rotates through an angle of 40°. Moreover, the Mg^2+^ coordination environment depends on the position of the ribose ring (figure 2*e*) and thus only exists in the ternary complex during catalytic turnover. The planes of the APDA and ribose ring are approximately parallel but do not overlap, thereby positioning the π-system of the pyrazole for nucleophilic attack at C1 of PRPP, and the Val138 side chain adopts the same position and conformation as that seen for Thr138 in the WT ForT/PRPP binary complex [32].

APDA makes a number of hydrogen bonding interactions that rationalize the biophysical data obtained for several of our ForT variants (table 1). For example, one of the carboxylate groups hydrogen bonds to the backbone NH and side chain of Ser174, which orders residues 172–179 of the protein in the T138V/APDA/MgPRPP ternary complex (figure 2*f*) and positions the adjacent pyrazole carbon 4.6 Å from C1 of PRPP. These residues are disordered in the WT ForT/PRPP binary complex [32]. This carboxylate also lies at a distance of 4.6 Å from the positively charged side chain of Lys263, which is bound to both Gln266 and a network of water molecules that engage the pyrazole carboxylate and Ser21. We note that replacing Lys263 by alanine gives a ForT variant that has low activity and reduced affinity for both PRPP and APDA. The other carboxylate of APDA hydrogen bonds to the side chains of Ser21 and Gln266, as well as two water molecules that form a network with the side chain of Glu211. These interactions order the loop at Leu206 (which is disordered in the WT ForT/PRPP binary complex (figure 1*b*), [32]) and they position the adjacent pyrazole carbon 5.1 Å from C1 of PRPP. We note that the S21A variant exhibits 10-fold and 100-fold reductions in *k*_cat_ and binding affinity for APDA, respectively (table 1). The Leu24 side chain is stacked against one face of the aromatic pyrazole ring. Remarkably, the extracyclic amine of APDA, which points towards Ile37 and Ser139, does not form any intermolecular hydrogen bonds. The pyrazole NH, however, appears to hydrogen bond to a water molecule that itself is positioned by a second hydrogen bond to the Glu211 side chain. This structure also explains the absence of a nucleotide product containing a C-N bond.

Of course, that the T138V variant is active, albeit with a 40-fold reduction in *k*_cat_, poses the question as to why we are able to trap the T138V/APDA/MgPRPP ternary complex. It is possible that the answer lies in the high concentration (100 mM) soak of both substrates in buffers that slow the reaction (electronic supplementary material, figure S5) immediately before freezing. This, coupled with the substantially decreased activity of T138V, might explain our ability to trap the ternary complex (figure 2*a*,*b*). Alternatively, the absence of the threonine sidechain might result in the misalignment of the two substrates in the crystal thereby preventing reaction and permitting observation of the complex.

**Figure 3.**
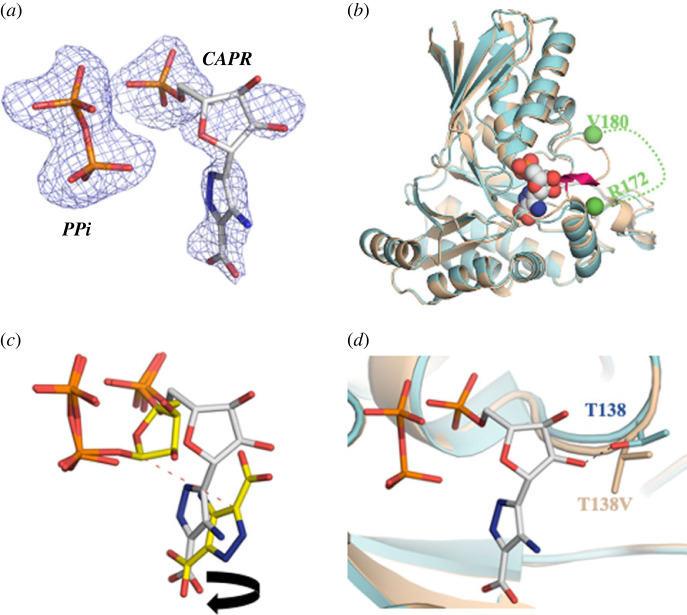
(*a*) Fo-Fc omit electron density map (blue mesh) contoured at 2.5*σ* identifies CAPR bound to the L24A protein structure. Carbon atoms are coloured white, other atoms as figure 2*a*. (*b*) The loop from R172 to V180 is disordered in the L24a/CAAPR/PPi ternary complex (cyan cartoon) but is otherwise unchanged from the ternary complex (wheat cartoon). The CAPR molecule is shown as spheres, coloured as in figure 3*a*. (*c*) A superposition using the proteins of the ternary and CPAR complexes reveals that the three phosphate groups of CAPR (atoms coloured as in figure 3*a*) and PRPP/APDA (atoms coloured as in figure 2*a*) occupy identical positions. The aromatic rings of APDA and CAPR occupy a similar volume, but their planes are flipped with respect to each other. Despite this, the retained carboxylate occupies a very similar position in both structures. (*d*) Thr138 alters its conformation to make a hydrogen bond (black dashed line) with the ribose in the CAPR structure.

We were also able to observe structural changes that take place as a result of catalysis by solving the X-ray crystal structure of the L24A/CAPR/PP_i_ product complex to 1.75 Å resolution in space group P4_1_2_1_2 (table 2). Clear electron density was observed for both products in the active site (figure 3*a*). The protein structure in the product complex is almost identical to that seen in the binary complex (RMSD of 0.41 Å over 319 overlapping C*_α_* atoms) [32]. The loop at Ser174 is disordered here similar to the binary complex and in contrast with the T138V/APDA/MgPRPP ternary complex (figure 3*b*). The location of free pyrophosphate (consequently its interaction with the protein) is identical to the pyrophosphate portion of PRPP in the ternary complex (figure 3*c*). The 5′-phosphate group of CAPR is likewise positioned identically to the 5′-phosphate PRPP in the ternary complex (figure 3*c*). These phosphate locations are also found in the binary complex [32]. The Mg^2+^-binding site has been eliminated, however, because the CAPR ribose ring occupies a position intermediate between those seen in the ternary and binary complexes (figure 3*c*). As a result, the 2′-OH and 3′-OH groups hydrogen bond to the Thr138 side chain and a water molecule, respectively (figure 3*d*). The heterocyclic ring of CAPR is ‘flipped’ relative to the APDA (figure 3*c*) and the extracyclic amine is within hydrogen bonding distance of a water molecule, but this water does not bridge to E211. We did refine a structure with the CAPR ring in the same orientation as seen for APDA but the fit to electron density was less satisfactory. The single carboxylate group of CAPR hydrogen bonds to the side chains of Ser21 and Gln266, in a similar manner to that seen for that cognate group in the T138V/APDA/MgPRPP ternary complex although the carboxylate atoms are shifted between 0.6 and 1.2 Å (figure 3*c*).

### 2.3. Computational modelling

**Figure 4.**
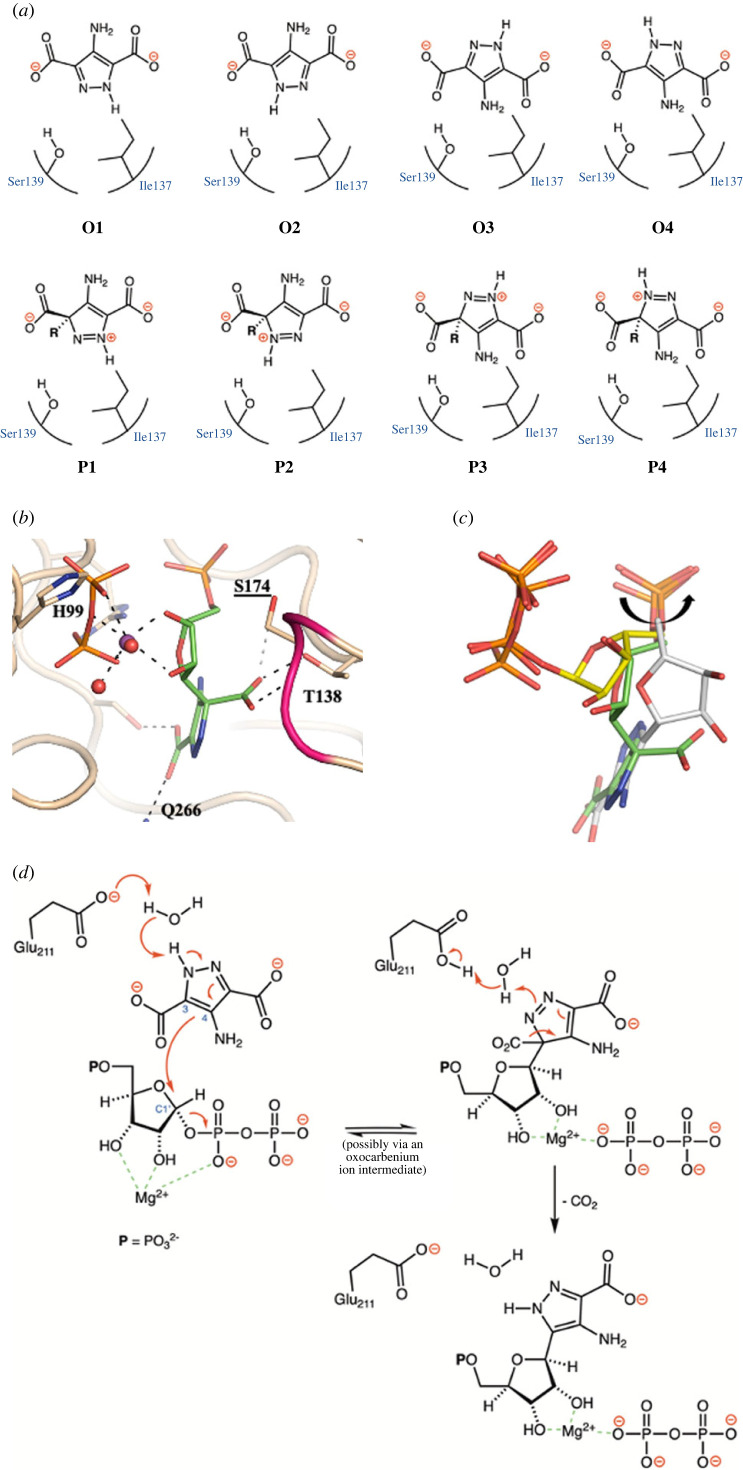
(*a*) Schematic representations of the cluster models used in the QC calculations. We considered both tautomers as well as the flipped orientation of the heterocycle. (*b*) The calculated intermediate (carbon atoms coloured green, other atoms coloured as in figure 2*a*) retains Mg^2+^ ion binding and is attached to the protein using the 5′-phosphate and retained carboxylate. (*c*) Superposition using the protein atoms of the complex with substrates (coloured as in figure 2*a*), calculated intermediate (coloured as in figure 4*b*) and CAPR (coloured as in figure 3*a*). The ribose pivots (shown as black arrow) around the 5′-phosphate during the reaction. (*d*) Proposed catalytic mechanism for ForT-catalysed C-C bond formation.

Access to the structure of the T138V/APDA/MgPRPP ternary complex permitted us to build an active site ‘cluster’ model [40] of the pre-decarboxylation intermediate proposed to link the WT ForT/MgPRPP/APDA Michaelis complex with the WT ForT/CAPR/PP_i_ product complex. In an effort to resolve the ambiguity concerning the pyrazole orientation in the active site and reactive tautomer of the heterocyclic ring, we built four cluster models of the Michaelis complex (O1–O4) (figure 4*a*) which were optimized using a localized, semi-empirical (PM7) description [41] after *in silico* reversion of Val138 to a threonine residue. All experimentally observed hydrogen bonds between the protein and both APDA carboxylates were maintained in each of the optimized cluster models of the Michaelis complex. These structures were then used to obtain cluster models of the four possible pre-decarboxylation intermediates (P1–P4) (figure 4*a*) by systematically decreasing the distance between C3 (APDA) and C1′ (PRPP) and optimizing the resulting models (electronic supplementary material, figure S9). As all other degrees of freedom were allowed to relax in the active site, the ribose-pyrophosphate (C1-O) bond lengthened as the pyrazole-ribose (C3-C1) bond was constrained to smaller lengths. This procedure, implemented in MOPAC2016, gave optimized structures in which the C1'-O bond was fully broken (2.9 Å), corresponding to PP_i_ release, when the C3-C1′ bond reached its optimum length. The heats of formation for all eight models were then computed using the semi-empirical PM7 parameterization [42] to give qualitative estimates of the activation energy barriers for this step. Assuming that each pre-decarboxylation intermediate resembles the transition state for this endothermic step, the lowest activation energy calculated for forming the pre-decarboxylation intermediate is when APDA adopts the orientation and tautomer (O4) seen in the T138V/APDA/MgPRPP ternary complex (table 3).

**Table 3.** Calculated heats of formation (kcal mol^−1^) for the active site cluster models of the WT ForT/MgPRPP/APDA ternary complex (O1–O4) and their associated pre-decarboxylation intermediates (P1–P4).

	O1	P1	O2	P2	O3	P3	O4	P4
Δ*H_f_* (kcal mol^−1^)	−17175	−17126	−17173	−17138	−17168	−17128	−17171	−17145
*E*_a_ (kcal mol^−1^)^a^		49		35		40		26

^a^Estimates of the activation energies are a lower bound and assume that the transition state for this endothermic step closely resembles the intermediate.

The optimized computational model of the pre-decarboxylation intermediate (P4) shows the reorientation of the departing carboxylate due to rehybridization of the adjacent carbon due to C-C bond formation between C1′ of the ribose and the pyrazole carbon (figure 4*b*). As a result, the carboxylate lies within hydrogen bonding distance of the Thr138 side chain and the backbone NH of Ser174. The lack of interaction with the Ser174 hydroxyl is consistent with the catalytic properties of the S174A variant (table 1). These QC calculations support the idea that the T138 side chain can stabilize the intermediate (and, by the Hammond postulate, the transition state for C-C bond formation) by hydrogen bonding. Experimentally, removing this potential hydrogen bond leads to a 40-fold reduction in *k*_cat_ for the T138V variant relative to ForT WT. On the other hand, the corresponding reduction is only threefold for the T138A variant (table 1). We suggest that a water molecule could enter the active site and ‘rescue’ the activity of the T138A variant by supplying a hydrogen bond to the carboxylate, an idea for which there is ample precedent [43]. The water that bridges the pyrazole NH and the Glu211 (figure 2*f*) is retained in the optimized structure, and the pyrophosphate leaving group is located within the binding pocket seen in the crystal structure of the L24A/CAPR/PPi product complex (figures 4*b*,*c*).

### 2.4. Substrate scope of WT ForT

**Figure 5.**
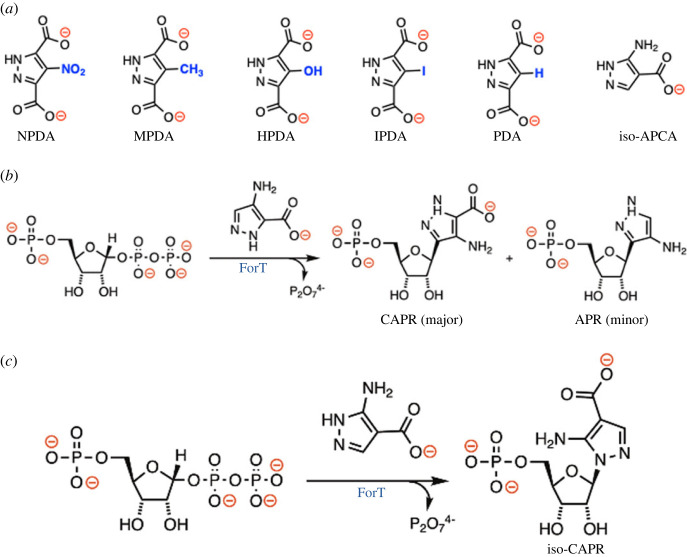
(*a*) Functionalized pyrazole-dicarboxylates used to explore the substrate scope of ForT. (*b*) ForT-catalysed reaction when APCA is the substrate [30]. (*c*) ForT-catalysed reaction when *iso*-APCA is the substrate.

We assayed a series of pyrazoles as ForT substrates in which the extracyclic amine was replaced by hydrogen (PDA), nitro- (NPDA), methyl- (MPDA) and iodo- (IPDA) groups (figure 5*a*; electronic supplementary material, table S1). None of these pyrazole-dicarboxylates proved to be substrates for the enzyme in our LC-MS assay (data not shown). We also confirmed the literature finding [30] that 4-hydroxy-1*H*-pyrazole-3,5-dicarboxylic acid (HPDA) is not a substrate for the ForT-catalysed coupling reaction even though HPDA binds to the ForT/PRPP complex with a *K*_D_ value of 37 µM (Li, unpublished). On the other hand, 4-amino-1*H*-pyrazole-3-carboxylate (APCA) can be coupled to PRPP by ForT to give two products containing a new C-C bond, albeit in less than 5% overall yield (figure 5*b*) [30]. In order to assess the importance of pyrazole substitution, we incubated 3-amino-1*H*-pyrazole-4-carboxylate (*iso*-APCA) (electronic supplementary material, table S2) and MgPRPP in the presence of ForT. LC-MS analysis showed that a new product had been formed with *m/z* of 340.1 (electronic supplementary material, figure S10), a value consistent with ForT-catalysed nucleotide formation and retention of the carboxylate substituent on the pyrazole ring. The ^1^H NMR showed signals for an aromatic CH group and the hydrogen connected to the ribose C1′, which was shifted downfield (5.74 ppm) relative to the signal for the cognate proton in the C-nucleotide CAPR (4.91 ppm) (electronic supplementary material, figure S11). Thus, the product was one of two possible C-N nucleotides. The ^13^C-^1^H HMBC NMR spectrum, however, shows an interaction between C5 of the pyrazole ring and C1'-H, as well as the absence of interactions between C3 and C1'-H (electronic supplementary material, figure S13). These data are consistent with the product being 5-amino-1-(β-D-ribofuranosyl)pyrazole-4-dicarboxylate 5′-monophosphate (iso-CAPR) (figure 5*c*) [44].

## 3. Discussion

The transformation catalysed by ForT is of chemical interest for a number of reasons. First, it is one of only a few examples in which C-C bond formation takes place via an electrophilic substitution of a small, aromatic heterocycle. Second, the reaction is regioselective despite the heteroaromatic substrate having multiple reactive nitrogen atoms that could react with the electrophilic carbon of PRPP. Third, ForT is capable of discriminating between the aminopyrazoledicarboxylic acid and its analogue in which the amine is replaced by a hydroxyl group; a remarkable feat given the steric and electronic similarities of the two molecules. The overall reaction is rendered essentially irreversible by coupling C-C bond formation to decarboxylation and PPi formation.

ITC measurements show that APDA binds to ForT only in the presence of PRPP (figure 1*c*), suggesting that the reaction likely follows an ordered bi-bi kinetic mechanism in which PRPP binds first and creates the binding site for APDA. Although Mg^2+^ is absent in these experiments in order to prevent catalytic turnover, we do not expect these conditions to alter the substrate binding order. Structural support for this mechanism is provided by the observation that the bound APDA molecule blocks PRPP from solvent in the T138V ternary complex (figure 2*f*). In addition, APDA-binding orders a loop due to main and side chain interactions of Ser174 with the carboxylate group of the substrate. This loop is disordered in the ForT/PRPP binary complex [32] and in the crystal structure of the L24A/CAPR/PP_i_ ‘product’ complex (figure 3*b*); the key APDA carboxylate group is absent in both of these structures. We propose this loop acts a gate keeper for substrate entry into, and product release from, the active site. Deleting the side chain of Ser174 reduces *K*_M_ for APDA only fivefold, leading to the conclusion that it is the hydrogen backbone to the backbone amide NH of Ser174 that is critical for substrate recognition and loop organization.

Our structural data also explain the regioselectivity of the reaction, which is associated with positioning only one carbon atom in the APDA ring at 4.6 Å from C1′ of PRPP with the correct orbital alignment for electrophilic substitution (figure 2*f*). A key observation from the structural data and the calculated model of the pre-decarboxylation intermediate is that the phosphate (PRPP, CAPR) and the pyrophosphate groups (PRPP, PPi) essentially stay fixed in position throughout the entire catalytic cycle (figure 3*c*). Similarly, the plane of the heterocyclic ring is preserved in the cluster model of the pre-decarboxylation intermediate P4 (figure 4*b,c*) and in the crystal structures (figure 3*c*). The π-system of the aromatic heterocycle interacts with the methyl substituents of the L24 side chain (figure 2*f*), consistent with a role for L24 in positioning APDA. This idea is supported by analysis of the L24A variant which reduces both the affinity of the enzyme for APDA and catalytic efficiency (table 1).

The retained carboxylate that is present in APDA crystal structure (figure 2*f*), CAPR crystal structure (figure 3*c*) and the calculated pre-decarboxylation intermediate (figure 4*b*) is anchored to the protein by direct contacts to Ser21, Gln266 and a bridging water network (figure 2*f*). (The precise arrangement differs slightly between the crystal structure (figure 3*c*) due to the flip of the plane of heterocycle.) Mutation of Ser21 profoundly weakens binding and catalysis while mutation of Gln266 leads to an unstable protein that cannot be kinetically characterized (table 1). Mutation of Lys263 which interacts with the water network also compromises catalysis (table 1). Compared to the binary complex [32], the ribose ring in the T138V ternary complex rotates to create the APDA-binding pocket (figure 2*e*), a movement that is accomplished merely by changes in the torsion angles connecting the ribose ring to the fixed phosphates at each end of PRPP; akin to a seat of a child's swing linked at both ends to a rigid frame. The ribose ring in the ternary complex adopts a C2′-endo conformation (figure 2*b*), which allows O2′ and O3′ and the pyrophosphate group to form a binding site for the Mg^2+^ ion (figure 2*b*). The two water molecules that complete the coordination sphere (figure 2*b*) are part of an extensive water network. The catalytically essential Mg^2+^ (or Mn^2+^) ion (electronic supplementary material, figure S3), probably stabilizes the C2′ endo conformation making PRPP more reactive, an arrangement first seen in HGPRT [39].

Combining the structural data from the ForT/PRPP binary complex [32], the T138V ternary complex, the model structure of the pre-decarboxylation intermediate, and the L24A product complex provides a picture of the substrate and protein motions that take place during catalysis. This analysis shows that it is the ribose that moves, swinging around both fixed ends as the binary complex converts to the ternary complex to CAPR. Thus, the change from ternary complex to the intermediate requires breaking the ribose-pyrophosphate (C1′-O) bond, thereby ‘untethering’ the ring at one end, which permits the ribose to rotate around the 5′-phosphate towards APDA (figure 3*c*). The ribose ring has become almost planar losing the C2′ endo pucker but retaining the Mg^2+^ ion (figure 4*b*). The carboxylate group of the intermediate that will be lost as CO_2_ likely forms a hydrogen bond to the side chain of Thr138 (figure 4*b*). The subsequent decarboxylation allows the ribose to continue its rotation towards the APDA site (figure 4*c*) and thus form a hydrogen bond between O2' and the hydroxyl group of Thr138 (figure 3*d*). The trajectory of the chemical reaction can therefore be thought of as the movements of ribose constrained by two anchors (figure 4*c*). In the binary to ternary complex conversion, these are the 5′-phosphate and pyrophosphate. In the ternary complex transformation to the intermediate, the 5′-phosphate anchors PRPP and the retained carboxylate anchors APDA (figure 4*c*). In the final step, decomposition of the intermediate to product, the 5′-phosphate and retained carboxylate act as tethers.

The order of the breakage of the C1'-O bond (PPi formation), decarboxylation of the heterocycle and formation of the new C-C bond is not known. Were the C1'-O bond to break as an isolated first chemical step to yield an oxocarbenium ion, we would have expected evidence of quenching of the ion by water. APDA is stable in our hands, a calculation of the substrate at the active site shows no evidence for decarboxylation. Further the extra cyclic hydroxyl, nitro and methyl substrate variants are inactive, suggesting that decarboxylation is unlikely to be the initial chemical step. Our QC calculations show the active site to be unchanged by formation of the pre-carboxylation intermediate P4 (figure 4*b*), this is consistent with decarboxylation occurring after C-C bond formation. We therefore conclude that the C-C bond formation most likely occurs in an S_N_2-like manner where both bond breaking and making at C1' occur at the same time, followed by decarboxylation.

Pulling the data together, allows us to propose the chemical mechanism of the enzyme. First, PRPP binds to create APDA-binding site and as APDA binds it both orders the loop at Ser 174 and forces the PRPP into an activated state (figure 4*d*). APDA and PRRP react in an S_N_2 like manner to form the pre-decarboxylation intermediate pivoting around the 5′-phosphate (figure 4*d*). Glu211 is essential for catalysis, and we suggest it acts as a general base to make APDA sufficiently nucleophilic (figure 4*d*). The formal positive charge in the heterocyclic ring of the intermediate triggers decarboxylation. The disordering of the protein loop which results from the release of CO_2_ permits the CAPR molecule to diffuse out of the active site, followed by the pyrophosphate group. The fact that the apparent K_M_ for APDA is smaller than its *K*_D_ value is evidence that product release is slow relative to substrate binding [45].

We have also considered the alternative orientation of the APDA seen in the CAPR complex (figure 3*c*). In principle, this reorientation might take place during the catalytic cycle, but we view this possibility as unlikely given the number of hydrogen bonds that would need to be broken prior during the rotation as part of catalysis. The simplest explanation is this orientation is a feature of the L24A variant used to obtain the structure. Indeed, the position of the pyrazole seen in the product complex would result in a clash with Ile37 in the native sequence [32]. This clash is avoided in the L24A variant because Ile37 adopts a new conformation that allows it to fill a pocket created by the absence of the Leu24 side chain. However, a very small manual adjustment of the positions of the reoriented APDA revealed that such a flipped conformation can avoid clashes, preserve very similar protein interactions, and position the APDA relative to PRPP to form the C-C bond (electronic supplementary material, figure S14). The extracyclic amine now in the water-filled pocket is 4.5 Å from the carboxylate of Glu211. Glu211 could act as a base directly by some further adjustment or by water bridge to active the APDA. Thus, the mechanism in figure 4*d* is preserved with only a trivial change (electronic supplementary material, figure S15).

QC calculations investigated how the two heterocyclic ring orientations might impact the activation energy for formation of the putative pre-decarboxylation intermediate. Making the assumption that the transition state resembles the pre-decarboxylation intermediate, these calculations suggest that the lowest barrier is associated with APDA adopting the orientation observed in the T138V ternary complex. More detailed QM/MM studies beyond the scope of this work will be required to unequivocally establish this conclusion.

Structural analysis predicts that a monocarboxylate analogue of ADPA should be bound by the pre-ordered Gln266 and Ser21 (figure 2*f*), not by Ser 174 which depends upon ordering of a loop. In this orientation, the monocarboxylate analogue would undergo electrophilic substitution *without* decarboxylation and regeneration of the aromatic π-system would take place via loss of a proton. The reaction of 4-amino-1*H*-pyrazole-3-carboxylic acid (APCA), however, leads to *both* CAPR and 4-amino-3-(β-D-ribofuranosyl)pyrazole 5′-monophosphate, a minor product that is entirely decarboxylated (figure 5*b;* electronic supplementary material, table S1) [30]. This mixture of products could imply that APCA is able to bind such that the carboxylate is anchored by Ser174. This would contradict our structure-based hypothesis about the need for carboxylate binding to Ser211 and Gln266. We reasoned that the isomeric compound 3-amino-1*H*-pyrazole-4-carboxylic acid (iso-APCA) (figure 5*c*) would be informative since our hypothesis predicts that iso-APCA will be anchored such that only one pyrazole nitrogen, the one not attached to the carbon linked to the carboxylate, would be correctly positioned. Consistent with our hypothesis we only observed the C-N nucleotide 5-amino-1-(β-D-ribofuranosyl)pyrazole-4-carboxylate 5′-monophosphate (electronic supplementary material, table S2). We propose that the formation of 4-amino-3-(beta-D-ribofuranosyl)pyrazole 5'-monophosphate occurs not from the carboxylate group of APCA binding to Ser174, but rather because the lack of the second carboxylate in the substrate allows more motion of APCA in the active site. As a consequence, the carbon adjacent to the pyrazolecarboxylate can come close enough to C1′ of PRPP to permit a competing reaction (electronic supplementary material, figure S15). Interestingly, the ability of ForT to turn over molecules such as APCA establishes that the loss of CO_2_ is not an indispensable driving force for C-C bond formation.

The formation of C-C bonds is a defining feature of biological chemistry, and ForT is capable of making both C-C and C-N bonds. Catalysis depends on Mg^2+^ ion and the high-energy pyrophosphate nucleotide (PRPP) (which has recently been shown to be made by prebiotic conditions [46]). The protein's role is limited to positioning the substrates and (perhaps) supplying a catalytic base. Intriguingly, the same positioning of substrates and use of MgPRPP is found in unrelated enzymes, hinting at perhaps an ancient origin of this biosynthetic chemistry.

## Data Availability

Coordinates and structure factor files have been deposited in the PDB with the following entries: 8AP0 for T138V/APDA/MgPRPP and 8AOZ for S24A/CAPR/PPi. Coordinates for models O1–O4 and P1–P4, as well as their associated input/output files, are available by clicking the following link: http://openmopac.net/Manual/Publications.html. Additional data are provided in the electronic supplementary material [47].
